# Experiences with complementary and integrative medicine in mental healthcare: a qualitative substudy of the PSYKIM project

**DOI:** 10.3389/fmed.2025.1629436

**Published:** 2025-09-02

**Authors:** Julia Siewert, Hannah Wackermann, Benno Brinkhaus, Michael Teut, Eva Jansen

**Affiliations:** ^1^Institute of Social Medicine, Epidemiology and Health Economics, Charité – Universitätsmedizin Berlin, Corporate Member of Freie Universität Berlin and Humboldt- Universität zu Berlin, Berlin, Germany; ^2^Institute of Social Medicine and Epidemiology the Brandenburg Medical School Theodor Fontane, Neuruppin, Brandenburg, Germany

**Keywords:** psychiatry, mental health, mental illness, qualitative research, patient experiences, complementary and integrative medicine

## Abstract

**Introduction:**

Complementary and integrative medical procedures (CIM) are commonly used in Germany, including for the treatment of mental health conditions. The aim of the study was to investigate how CIM is used and perceived in outpatient mental healthcare settings.

**Methods:**

This qualitative interview study was conducted as part of the PSYKIM cross-sectional project. Twenty participants (15 women, 5 men; mean age 37.5 years, range 19–64) were recruited from a larger survey sample. Semi-structured telephone interviews were used to explore participants' experiences with CIM therapies in the context of mental healthcare. Interview data was analyzed using qualitative content analysis within a constructivist paradigm.

**Results:**

The most frequently used CIM therapies were yoga, acupuncture, meditation, art therapy, and progressive muscle relaxation (PMR). Thematic analysis identified four overarching experiential dimensions across therapies: (1) emotional regulation and inner calm, (2) individual prerequisites and ambivalent effects, (3) creative expression and emotional processing, and (4) the influence of therapeutic setting. CIM therapies were experienced in highly heterogeneous ways. While many interviewed participants reported symptom relief, improved wellbeing, and enhanced self-awareness through CIM therapies, others described frustration, emotional distress, or a lack of effect. Overall, experiences were highly individualized and influenced by factors such as personal readiness, the therapeutic environment, and group dynamics.

**Conclusion:**

The highly heterogeneous ways in which patients with mental health conditions experience CIM therapies underscore the need for individualized implementation, professional guidance, and open communication about both benefits and potential risks. Future research should investigate how multimodal interventions that combine CIM with conventional treatments can be individually tailored and contextually adapted to improve mental health outcomes.

**Trial registration:**

This study has been registered in the German Clinical Trial Registry with trial ID DRKS00032426 on 08.08.2023.

## Introduction

Complementary and integrative medicine (CIM) refers to a variety of medical systems and therapeutic approaches that are commonly used alongside, or as an alternative to, conventional healthcare in Europe (1). The definition of CIM in this study aligns with the World Health Organization, which describes complementary medicine as additional healthcare practices not part of a country's mainstream medicine, and integrative medicine as an interdisciplinary, evidence-based approach combining biomedical and traditional and/or complementary medical knowledge, skills, and practices (2). CIM approaches such as herbal medicine, acupuncture, Traditional Chinese Medicine, Ayurveda, and homeopathy are widely used by individuals with depression, anxiety, and other mental health conditions (3–9). Given that the lifetime risk of developing a psychiatric disorder is estimated at around 50% (10), the integration of CIM into mental healthcare is becoming increasingly relevant. Both patients and healthcare providers are increasingly incorporating CIM into the treatment of mental health conditions (11, 12). A 2019 survey conducted in Swedish mental health facilities, including outpatient, day, and inpatient clinics, found that 62% of patients used CIM to manage symptoms such as anxiety, sleep disturbances, and depression (13).

Worldwide studies have shown that CIM plays an important role in the care of individuals with mental health conditions (11–18). Key reasons for its use include patients' own initiative in coping with their condition, perceived symptom relief, treatment satisfaction, reduced use of pharmaceutical medication, and the desire to avoid side effects (7, 13).

A Norwegian cross-sectional study involving 12,982 patients found that individuals with anxiety and/or depression who used psychiatric outpatient services were also more likely to consult CIM providers, suggesting that many patients perceive CIM as a parallel or supplementary form of care for mental health concerns (17).

A 2018 survey found that an estimated 3.6% of people worldwide with a mental disorder had contact with CIM in the previous 12 months (based on data from 2001 to 2012). In high-income countries, the rate was approximately twice as high at 4.6% compared to low- and middle-income countries (15). Additionally, satisfaction with CIM among individuals who received both CIM and conventional care was reported to be high (15).

A 2024 survey conducted in Germany with 4,065 participants (52% female, 48% male, 0.4% diverse) found that 70% had used CIM at some point in their lives. According to the authors, these results from a representative online sample suggest that the use of CIM in Germany remains consistently high (19). The extent to which CIM is used in outpatient psychiatric and psychosomatic care in Germany, and for what purposes, remains largely unknown, partly due to the heterogeneity of existing studies, which often focus on general or somatic populations rather than psychiatric contexts (20–22).

The aim of the qualitative component of the PSYKIM cross-sectional study (Utilization of Complementary and Integrative Therapies in Outpatient Mental Health Care in Germany: a Cross-Sectional Study) was to explore how individuals with mental health conditions experience and evaluate the use of CIM in outpatient care in Germany.

Specifically, the qualitative component of the PSYKIM study aimed to:

• Explore how individuals with mental health conditions subjectively experience CIM therapies in outpatient settings.
• Identify perceived emotional, cognitive, and physical effects across different CIM therapies.
• Understand contextual factors such as therapeutic setting, individual readiness, and social dynamics that influence these experiences.
• Examine how participants make sense of both beneficial and adverse outcomes in relation to coping and overall wellbeing.

## Methods

### Study background and participant recruitment

This qualitative sub-study was part of the PSYKIM project. In total, 770 adult patients with a diagnosed mental health condition were surveyed online and in outpatient care centers within the PSYKIM cross-sectional study in both urban and rural areas of Berlin and Brandenburg. The results of the quantitative component are currently under analysis and will be published separately.

In contrast to the quantitative component, the qualitative sub-study focused on participants' subjective experiences with CIM therapies in the context of mental healthcare. Participants for the qualitative study were recruited from the larger PSYKIM cross-sectional sample. Recruitment was carried out via multiple public channels, including advertisements on public transportation, newspaper announcements, flyers at general practitioners' offices, and information provided on the website of the conducting institute. To be eligible, participants had to be at least 18 years old, of any gender, and have a self-reported mental health diagnosis. Exclusion criteria included lack of sufficient German language skills, cognitive impairments preventing informed participation, current full inpatient psychiatric, psychotherapeutic or psychosomatic treatment, or declared unwillingness to continue study participation. Participants received a €20 incentive for completing the interview. From the pool of consenting individuals in the main study, 20 participants were randomly selected for qualitative interviews.

### Sampling strategy

Participants were sampled to capture a range of experiences with CIM therapies among people with various mental health conditions. From the pool of volunteers who had provided informed consent, 20 participants were randomly selected. Random selection was applied to reduce selection bias and to allow for the inclusion of diverse perspectives, while maintaining the exploratory character of the study. The resulting sample was heterogeneous with respect to gender, age, and therapy experience.

All interviews were conducted via telephone between March and August 2024. Interview durations ranged from 11 to 58 min, with an average of 20 min and 5 s.

### Time and conduct of interviews

Interviews were conducted by a trained qualitative researcher (HW), with no prior relationship to the participants. Before each interview, participants were informed about the study's aims and the role of the interviewer. All interviews followed a semi-structured format guided by a thematic interview guide (see Appendix 1), which included topics such as participants' prior experiences with natural healing methods, perceived effectiveness and side effects, contextual factors influencing therapy outcomes, and their willingness to use CIM again.

### Interview data collection and protection

All interviews were audio-recorded digitally and pseudonymized. Recordings were encrypted immediately after the interview and deleted from the recording device. Transcription was carried out verbatim by a professional transcription service. Transcripts were anonymized, and data storage complied with applicable institutional and legal data protection standards. Transcripts were not returned to the interviewed participants for comments or corrections. Participants did not provide feedback on findings.

### Interview content and guide development

The semi-structured interview guide was developed based on literature review, expert consultation, and study objectives. Key areas included therapeutic satisfaction, emotional and physical responses, perceived changes in symptoms and wellbeing, and contextual experiences such as therapist interaction and setting. Participants were also asked about any perceived risks or negative outcomes and whether they would consider using CIM again.

### Interview data analysis

Interview data was analyzed using qualitative content analysis as described by Hsieh and Shannon (38), following a constructivist paradigm. This approach assumes that knowledge is co-constructed through language and shaped by individual experience and social context. The analysis aimed to capture participants' subjective meanings and interpretive patterns related to their use of complementary and integrative medical (CIM) procedures.

A combination of inductive and deductive coding was applied: inductive codes emerged directly from the interview data, while deductive categories were informed by the study's research aims and the interview guide. Initial coding involved identifying meaningful units in the transcripts, which were grouped into descriptive categories. These categories were iteratively refined through constant comparison to develop overarching themes that reflect patterns and variations of experience across participants.

This analytic framework enabled the development of cross-cutting experiential themes that emerged across CIM therapies. The analysis focused on identifying shared patterns, ambivalent dynamics, and contextual influences shaping participants' experiences. This approach facilitated a more integrated understanding of how CIM was perceived and evaluated in the context of mental healthcare. All coding and theme development were conducted using MAXQDA^®^ 2024 software, and analytic decisions were regularly discussed within the qualitative research team to ensure reflexivity and consistency.

## Results

### Participant characteristics

Twenty participants (15 female, 5 male; age range 19–64 years, mean age 37.5) took part in semi-structured interviews (see Table 1). The most common illness across participants was depression. The mean duration of self-reported illness was 15.2 years. When they were asked to assess how severe their illness was, the average self-reported severity of illness corresponded to a level between mild and moderate.

**Table 1:** Participant characteristics.

**ID**	**Age group**	**Gender**	**Profession**	**Illness**	**Self-reported duration of illness in months**	**Self-reported severity**
1	18–25	Female	Job seeking	Moderate depressive episode	4	Moderate, tendency to mild
2	26–35	Female	Nursing assistant	Severe depression and borderline	1	Moderate, tendency to severe
3	36–45	Male	Farmer	Multiple dependencies	22	Mild
4	56–65	Male	Retired	Generalized anxiety disorder, moderate depression	38	Mild
5	18–25	Female	Social assistant	Borderline	12	Moderate
6	36–45	Female	MFA (Medical Specialist Assistant)	Moderate depression, panic attacks, anxiety disorder	1	Mild
7	18–25	Female	Job seeking (unable to work)	Borderline, behavioral disorder, atypical autism	3	Moderate
8	26–35	Female	Job seeking	Borderline, anxiety disorder, depressive disorder	7	Severe
9	36–45	Female	Educator	Depression, trauma	20	Moderate
10	26–35	Female	Science and event management	Anxiety disorder	25	Mild
11	56–65	Female	Healthcare	Depression, panic attacks, anxiety	21	Moderate
12	36–45	Female	Educator	PTSD and/or depression	20	Mild
13	46–55	Male	Social worker	Addiction, depression, anxiety disorder	24	Moderate
14	46–55	Female	Piano teacher	Depression, personality disorder	8	Mild
15	18–25	Female	Job seeking	Eating disorder, depression	15	Moderate
16	56–65	Male	Marketing manager	Bipolar disorder	8	Mild
17	18–25	Female	MTLA (Medical Technical Labor Assistant)	Depression	15	Mild
18	26–35	Female	Employed	Chronic depression	4	Moderate
19	36–45	Female	Tax clerk	Moderate depressive episode	13	Moderate
20	26–35	Female		Moderate depressive episode	10	Moderate
Mean (Range)	37.5 (19–61)				15.2 (1–38)	

### Use of complementary and integrative medicine (CIM)

The interviewed participants described using a wide variety of complementary and integrative medicine therapies to support their mental health. The most frequently mentioned individual therapies were yoga (9 mentions), acupuncture (8), meditation (7), progressive muscle relaxation (5), and art therapy (5). To improve clarity and comparability, all named therapies were grouped into broader conceptual categories (see Table 2). The most commonly used categories were Meditation & Relaxation (e.g., meditation, PMR, autogenic training), Mind–Body Practices (e.g., yoga, Tai-Chi), and Traditional Systems (e.g., acupuncture, traditional Chinese medicine, homeopathy).

**Table 2:** Grouped CIM therapies by category and frequency.

**CIM category**	**Included therapies**	**Mentions (*n*)**	**Most frequently used therapies**
Meditation and relaxation	Meditation, PMR, autogenic training	13	Meditation (7)
Mind-body practices	Yoga, Tai-Chi	10	Yoga (9)
Traditional systems	Acupuncture, traditional Chinese medicine, homeopathy	10	Acupuncture (8)
Nutritional approaches	Dietary changes, supplements, oils, and herbs	9	Herbal medicine (5)
Creative therapies	Art therapy, diary	7	Art therapy (5)
Supportive techniques	Self-help groups, apps, walks, dog	6	Apps, walks (~2 each)
Manual therapies	Osteopathy, chiropractic, massage ring	4	Osteopathy (~2)

Other categories included Nutritional Approaches, Creative Therapies, Supportive Techniques, and Manual Therapies, although these were reported less frequently. General physical activity (e.g., sports), though frequently mentioned, was excluded from analysis as it does not fall under standard definitions of CIM. This structured categorization of therapies provides a foundation for the thematic interpretation of participant experiences presented in the following section.

### Thematic analysis of participant experiences

In the qualitative analysis of interview data, we explore the subjective experiences reported by participants with frequently used (CIM) therapies for mental health support, including yoga, acupuncture, meditation, art therapy, and progressive muscle relaxation (PMR). The interview data revealed overarching experiential themes that emerged across therapies. Based on participants' descriptions, four key experiential dimensions were identified: emotional regulation and inner calm, individual prerequisites and ambivalent effects, creative expression and emotional processing, and the influence of the setting and therapeutic framing. This approach aims to highlight shared experiential patterns and contextual factors that shaped participants' perceptions of benefit or burden—regardless of the specific CIM method used.

A summary of the reported experiences with each individual CIM therapy, including perceived positive and negative effects as well as relevant contextual influences, is provided in Table 3 to complement the thematic interpretation presented below.

**Table 3:** Reported experiences with selected CIM therapies (*n* = 5–9 per method).

**CIM therapy**	**Positive impacts**	**Negative impacts**	**Relevant contextual factors**
Yoga	Relaxation, improved wellbeing, sleep, body awareness	Pain, frustration, emotional deterioration, boredom	Need for professional guidance; group discomfort
Meditation	Calming thoughts, emotional regulation, improved focus	Flashbacks, loneliness, nightmares, ineffectiveness	Importance of environment and mood
Acupuncture	Symptom relief, life changes, sleep, physical relaxation	No effect, bleeding, pain, disbelief in method	Relaxing atmosphere; relationship to staff
Art therapy	Emotional processing, self-exploration, creative activation	–	–
PMR	Relaxation, stress reduction, improved body awareness	Short-lived effect, difficulty concentrating, tension in groups	Preference for individual setting; group dynamics

**1. Emotional regulation and inner calm**

A central benefit reported by the interviewed participants was the ability of CIM to support emotional regulation and foster a sense of inner calm. This was most commonly associated with meditation, yoga, PMR, and acupuncture. The interviewed participants described these practices as ways to counteract inner tension, calm distressing thoughts, and regain control over emotional states.

Meditation and PMR, in particular, were seen as facilitating relaxation and reducing psychological strain:

“So my pulse goes down and I notice how my body becomes softer again, my shoulders also become calmer, my thoughts become quieter, and the fears recede, exactly.” (P6)

“It has definitely given me back quality of life, but there's a difference when you feel tense and stressed all the time. That's super exhausting. When you then realize that you actually feel lighter and softer afterwards, that's definitely a good deal better.” (P10)

PMR, like meditation, was perceived by some as a tool to release inner tension and restore a sense of control.

Yoga was also experienced as supportive for managing stress and improving wellbeing:

“I also believe it helps with falling asleep. And I have the feeling that the tension and stress that arises from being sick […] needs to be released somewhere.” (P12)

In the context of acupuncture, the interviewed participants noted a gradual shift toward a more relaxed state, especially with repeated sessions. The cumulative effect of practice and routine was highlighted:

“The more often you do it, you somehow become more relaxed, but I can't really say why.” (P2)

These accounts suggest that, across therapies, many participants found CIM to be a way of restoring inner balance—emotionally, physically, and mentally.

### Individual prerequisites and ambivalent effects

Despite many positive reports, participants also described ambivalence and variability in their experiences. Some methods only helped under specific conditions, while others triggered frustration, psychological discomfort, or physical strain. Participants' accounts highlight the non-linear and highly individualized nature of their responses to CIM.

For example, yoga was seen as effective only when participants were in the “right” emotional state or had realistic expectations:

“Sometimes it doesn't help me find calm at all.” (P10)

Meditation, while calming for some, led others to feel overwhelmed or emotionally destabilized:

“Well, I struggle with flashbacks or inner images, and the sinking into that and not being really in reality doesn't do me good, it happens faster then.” (P12)

Unmet expectations or delayed effects could lead to disappointment and discouragement:

“Yeah, it's kind of frustrating because you always hope that it will get better soon, and then it doesn't seem to work out somehow, and that's really challenging.” (P9)

These examples reveal that CIM therapies are not inherently benign. Their subjective effectiveness appears to be contingent on individual psychological readiness, the therapeutic environment, and the ability to manage inwardly directed attention.

### Creative expression and emotional processing

Creative approaches such as art therapy were highlighted as particularly helpful in enabling emotional expression, distancing from distressing content, and regaining personal agency. Art therapy was not only used to express difficult emotions but also to transform them into something tangible and manageable.

One participant described how the visual representation of emotions created space for reflection and emotional clarity:

“While drawing or pottery, I was able to think about the problems but could view them a bit more distanced, without my entire emotional world overwhelming me.” (P9)

For others, art therapy reactivated creative potentials and was perceived as a process of self-recovery:

“After a few weeks of art therapy, I have really regained access to artistic things […] it really opens up entirely different avenues.” (P5)

These Participants' experiences suggest that expressive, non-verbal therapies may provide therapeutic value beyond symptom relief—by fostering self-exploration, identity reconstruction, and emotional resilience.

### Influence of the setting and therapeutic framing

The setting in which CIM was practiced played a decisive role in shaping perceived effects. Participants repeatedly referred to factors such as social proximity, professional guidance, emotional atmosphere, and physical environment. These conditions could either enhance or diminish therapeutic impact.

A calm and supportive environment—especially in acupuncture—was reported as essential for the experience of benefit:

“During that time, I could actually relax relatively well, which was difficult for me at that time.” (P17)

In contrast, the lack of personal space or guidance during group sessions, particularly in yoga or PMR, could provoke discomfort as one participant felt uncomfortable due to the proximity of the other participants during the yoga exercises. A further participant had general difficulties performing relaxation exercises in a group with other people. These observations highlight that CIM is not experienced in isolation, but always embedded in a social and structural context. The therapeutic setting—its safety, structure, and interpersonal quality—emerged as a cross-cutting factor that could either enable or hinder therapeutic experiences.

## Discussion

### Summary of results

The qualitative analysis of interview data revealed that participants' experiences with CIM for mental health support were highly individualized and shaped by a dynamic interplay between personal, emotional, and contextual factors. Across therapies, CIM was often described as a means of achieving emotional regulation and inner calm, offering participants relief from stress, anxiety, or inner turmoil. However, these effects were not uniform: ambivalence, disappointment, and even psychological worsening were reported, particularly when interventions lacked adequate support or did not align with participants' emotional readiness.

Creative and expressive therapies such as art therapy enabled participants to process emotions non-verbally and reconnect with personal resources, while practices like meditation or yoga required favorable internal and external conditions to be experienced as beneficial. The perceived effectiveness of CIM was strongly influenced by the therapeutic setting, including group size, emotional safety, and professional guidance. These findings underscore the importance of considering both individual and contextual variability when implementing CIM in mental healthcare and suggest that one-size-fits-all approaches are unlikely to meet the complex needs of this population.

### Creative expression, emotional processing, and self-recovery through art therapy

The interviewed participants consistently emphasized creative approaches, particularly art therapy, as valuable for emotional processing and personal development. These findings align with existing qualitative research on art therapy, which supports its benefits for patients with mental health disorders and recognizes it as an integral component in clinical practice (24, 25). The interviewed participants emphasized that art therapy facilitated not only the expression of challenging emotions but also their transformation into tangible and manageable forms. Specifically, the experience of achieving emotional distance from distressing content through creative activities like drawing or pottery allowed participants greater reflection and emotional clarity. Similar findings were described by Moser (24) in a qualitative study involving patients with psychosomatic disorders, which showed that art therapy not only provided a means for expressing difficult emotions but also strengthened self-confidence and enhanced coping skills for everyday life. Moreover, participants described how art therapy reactivated creative potentials, supporting processes of self-exploration and identity reconstruction. This perspective complements previous studies, such as Plecity (25), who also identified art therapy as helpful for becoming aware of emotional difficulties and actively managing them.

These qualitative experiences are further supported by quantitative studies. For instance, a systematic review by Xu et al. (26) demonstrates the effectiveness of creative arts therapies in reducing symptoms of depression and anxiety and improving general wellbeing. Overall, this discussion illustrates that art therapy can make a meaningful contribution to mental health by enabling the transformation of emotional experience through creative expression. Rather than offering symptom relief alone, it appears to foster emotional resilience, deepen self-reflection, and support the reconstruction of identity and personal meaning.

### From relaxation to regulation: the role of CIM

The findings of our study suggest that many participants experienced CIM therapies as supportive tools for emotional self-regulation and regaining emotional stability. Across therapies, body-based practices, particularly when practiced regularly, were perceived as helpful in calming intrusive thoughts, reducing internal tension, and achieving a state of calm and stability. This aligns with qualitative findings on yoga practice, which indicate that regular and personally meaningful engagement can facilitate not only symptomatic relief but also deeper experiential change (27). Capon et al. (28) demonstrated, in a qualitative interview study on anxiety and depression, that integrating elements of therapeutic yoga, such as breathwork, body-focused mindfulness, and values-based reflection, can effectively support psychotherapeutic processes by enhancing emotional regulation and promoting behavioral change. These findings are consistent with reports from our study, where participants emphasized the importance of mindfulness, body awareness, and cognitive focus in managing symptoms.

Building on these findings, the role of values-based reflection and meditation in fostering emotional change is further supported by a qualitative study by Gross et al. (29). In this study, female participants in the Meditation-Based Lifestyle Modification program reported deeper emotional integration and a stronger alignment with personal values. In particular, meditation and value-oriented reflection were perceived as facilitating profound shifts in emotional experience and self-perception (29).

Our interview data similarly indicate that CIM may not only trigger short-term relaxation responses but also support longer-term processes of emotional stabilization, particularly when the practices are experienced as personally meaningful and applied consistently. These findings suggest that CIM may foster self-awareness through body-based attention, support emotional processing by reducing internal arousal, and, in some cases, facilitate reflection on personal values. Such approaches may offer an experiential bridge between internal insight and embodied emotional regulation. Stabilizing effects were particularly evident among participants who engaged with these practices regularly and integrated them into their daily routines.

### Individualized responses to complementary therapies in mental health: between benefit and burden

The findings of this study highlight the ambivalent and highly individualized effects of complementary therapies, such as yoga, meditation, progressive muscle relaxation, and acupuncture, in the treatment of mental health disorders. Consistent with previous research, therapeutic outcomes appear to be shaped by individual factors, especially patients' expectations and personal needs. In a qualitative study, Richardson (30) found that people seeking complementary therapy often do so with specific hopes regarding symptom relief, holistic care, and alignment with personal values and preference.

In a randomized controlled mixed-methods study, Kinser et al. (31) investigated the effects of gentle Hatha yoga in women with major depression. Participants reported various benefits, including enhanced self-awareness, reduced rumination, improved emotional regulation, and a greater sense of self-care and social connectedness (31). These outcomes align with our participants' reports, particularly regarding improvements in mindfulness, cognitive focus, and body awareness. However, unlike Kinser et al., some of our participants also described frustration over delayed effects and discomfort in group settings. These differences may reflect variations in sample characteristics, delivery formats, or contextual factors, underscoring that therapeutic benefits are not universally experienced.

Both our findings and those of Capon et al. (28) in a qualitative study with cognitive-behavioral therapy integrating elements of therapeutic yoga for anxiety and depression, indicate considerable individual variability in the perceived effectiveness of body-based interventions. Psychological readiness, current mood, and treatment expectations emerged as key factors shaping participants' experiences (28).

A comparable pattern emerged with mindfulness meditation. In a qualitative study embedded within a mixed-methods evaluation of a mindfulness-based cognitive therapy program, Boggs et al. (32) reported that individuals with residual depressive symptoms experienced enhanced emotional regulation and greater cognitive awareness. Participants also noted practical challenges in maintaining home practice and translating skills into daily life, particularly around managing time and adapting exercises to their routines (32). Our findings reflect this ambivalence, with reports ranging from increased self-awareness to heightened rumination or discomfort during meditation. Progressive muscle relaxation, though often beneficial, also produced varied reactions, some participants experienced calm, others anxiety. These observations suggest that mind–body interventions can be psychologically demanding if not individually tailored and professionally supported.

These findings highlight that CIM therapies are not universally effective but vary depending on individual and contextual factors. While some patients benefit, others report distress or limited improvement. This underlines the importance of individualized, psychologically supported use of such methods. Clear guidance, realistic expectations, and professional monitoring are essential to prevent adverse reactions and enhance integration into mental healthcare.

### Importance of the setting

The analysis of participants' experiences highlights the central role of the therapeutic setting in shaping perceived outcomes. Group dynamics, emotional atmosphere, and interpersonal interactions emerged as key contextual variables influencing the perception of CIM treatments.

A controlled intervention study on group therapy for complicated grief demonstrated how differently social contact and attention can be experienced. Social interactions within group settings and the prevailing emotional mood significantly influence therapeutic outcomes. The quality and source of social support, whether from friends, family, or a special person, can have varying effects, as indicated in findings by Ogrodniczuk et al. (33), which suggest differential outcomes depending on the source of support and therapy context. These findings align with a review by Webber et al. (34), which emphasized that the quality, not quantity, of social relationships and the experience of group belonging are critical factors in psychosocial interventions for individuals with mental illness. Group settings fostered social identity and emotional wellbeing, underscoring the subjective and context-dependent nature of social influences in therapy.

In addition to relational aspects, contextual features of the therapeutic setting also influenced how participants experienced the intervention. Our study revealed that environmental aspects such as group size, time of day, or elements like music were associated with perceived benefits. In a qualitative study with adolescents with cancer, participants described music as soothing and comforting, and noted that it changed the treatment environment in a meaningful way (35). Zollman and Vickers (36) provide a narrative overview of key patient-related aspects in complementary medicine, emphasizing the importance of a calm atmosphere, sufficient time, personal attention, physical contact, and continuity of care. These features were not perceived as curative in themselves, but as facilitating emotional relief, a sense of safety, and improved patient satisfaction with care. CIM can foster emotional resilience, instill renewed hope, and help patients regain a sense of meaning and purpose (36).

This is also reflected in a qualitative study by Hopton et al. (37), embedded within an RCT, which explored patient experiences with acupuncture and counseling for depression, including comorbid pain. Participants emphasized individualized treatment, active participation, and a strong therapeutic alliance as central to both emotional and physical improvements. Moreover, in our study, participants emphasized that practices such as PMR, meditation, or acupuncture are most effective when embedded in a multimodal, therapeutically supported setting, rather than applied in isolation.

Consequently, the setting should be more individually tailored in order to optimize outcomes patient experiences, such as by offering smaller group formats, integrative elements or the possibility of conducting therapies in a private and safe setting.

### Strengths and limitations

To date, there have been very few scientific studies on the use of CIM by people with mental illness. A methodological advantage lies in the targeted recruitment of participants. The online survey was not distributed via relevant platforms for complementary medicine, but focused on mental illness. This enabled a more thematically differentiated sample and reduced potential bias due to a selectively complementary medicine-orientated group of participants. In addition, participants in the qualitative study were recruited from both the online survey and the on-site survey (e.g., in clinics, day clinics or outpatient practices). This allowed people to be included in the study who were unable to take part in an online survey due to their illness, the severity of their illness, or a lack of digital resources.

Nonetheless, several limitations should be considered. First, the sample size was relatively small (*n* = 20), and the on-site recruitment was limited to two federal states (Berlin and Brandenburg), which restricts geographical representativeness. However, as a qualitative study, the aim was not statistical generalizability but an in-depth exploration of subjective experiences. Still, limited regional scope should be considered when assessing transferability. Second, most participants in the qualitative interviews had affective or anxiety-related disorders, while more severe or less common diagnoses, such as psychotic disorders (e.g., schizophrenia), were not represented. As such, the findings may not capture the full spectrum of experiences with CIM across different diagnostic groups, and transferability to other clinical populations remains limited.

Third, the study did not include comparative interview data on participants' experiences with conventional therapies (e.g., medication, psychotherapy). While this aligns with the study's aim to explore CIM-specific experiences, it limits interpretive scope regarding the perceived role, added value, or relative effectiveness of CIM in relation to standard care. Fourth, the sample showed an overrepresentation of individuals from healthcare and service professions, which may reflect a higher familiarity with therapeutic settings and potentially greater openness to CIM. This could have influenced the way participants framed their experiences. Fifth, interview durations varied considerably (11–58 min), possibly affecting interview data depth and consistency. Shorter interviews may have yielded less detailed responses, although all contributed meaningfully to thematic development.

Sixth, transcripts were not returned to participants for member checking, which may limit the ability to verify or clarify interpretive accuracy. Reflexive coding and regular team discussions were employed to mitigate this, but direct participant feedback might have added further validity. Finally, the implementation of the study across multiple sites involved substantial logistical and personnel resources. This should be considered in the planning of future multi-site qualitative research on CIM in mental health contexts. While these limitations constrain generalizability, they are inherent to the qualitative methodology and are offset by the study's depth of insight.

### Future research

Future research should systematically address the following areas to advance the understanding and implementation of complementary and integrative medicine:

investigate how socio-demographic factors (e.g., age, gender, education, socioeconomic status) influence the utilization and acceptance of CIM approaches for example through cross-sectional surveys combined with qualitative interviews to explore subgroup-specific experiences.

Examine the impact of personal attitudes and beliefs of healthcare professionals, including physicians and therapists, on the recommendation, uptake, and evaluation of CIM interventions, using qualitative interviews and standardized attitude measures to identify key facilitators and barriers.

Explore how patients in Germany perceive the interplay between conventional medical treatments and CIM, with particular attention to expectations, experiences, and perceived benefits or challenges, which could be studied through in-depth interviews or longitudinal mixed-methods designs.

## Conclusion

The therapeutic use of CIM approaches, as described by the interviewed participants, was experienced in highly individualized ways, producing both beneficial and adverse effects. This variability highlights the need to provide patients with mental health conditions with clear information about possible CIM methods, and to select and apply CIM approaches in a personalized, careful manner. In particular, multimodal interventions like practices such as yoga, meditation, and PMR seems to have beneficial aspects and appear to be sensitive to contextual factors, including group size, time of day, and environmental setting. These findings emphasize the importance of tailoring interventions to individual needs and providing therapeutic support when negative experiences arise. Future research should investigate how multimodal interventions that combine CIM with conventional treatments can be individually tailored and contextually adapted to improve mental health outcomes.

## Data Availability

The original contributions presented in the study are included in the article/Supplementary material, further inquiries can be directed to the corresponding author.
