# Supplementary material for: Experiences with complementary and integrative medicine in mental healthcare: a qualitative substudy of the PSYKIM project
Source: Front Med (Lausanne). 2025 Sep 2;12:1629436. doi: 10.3389/fmed.2025.1629436 (PMC12436651; doi:10.3389/fmed.2025.1629436)
Supplement: Supplementary file 1 [file Data_Sheet_1.pdf]

Psykim Study

Interview guide for participants

Version 02, dated 25.01.2024

The interview will take place after participation in the cross-sectional study.

Participants will be contacted by phone or email.

Questions beginning with a number are core questions.

Indented questions beginning with a letter are possible additional questions.

Introduction

I am very glad that you have agreed to participate in an interview.

This is a safe and anonymous space, and you can terminate the interview at any time.

I will ask you open questions, I know this can be challenging, and you are welcome to share anything that comes to mind.

Do you have any questions before we start?

Basic Information

Age:

Gender:

Occupation:

Duration of illness in months:

Subjective severity of your main psychological complaint: mild – moderate – severe

Opening questions:

1. What psychological disorder do you have?
2. What complementary medical treatments (such as natural remedies, acupuncture, relaxation techniques, nutrition, and others) have you used? This includes anything that is not related to medications, behavioral therapy, or psychoanalysis.

3. What was the most important aspect of these treatments for you, the one you liked the most? (Start with this question)

Satisfaction with Therapy Application

4. How did you experience the therapies yourself?

a. What did you like, and what did you like less?

b. Is there a reason why?

c. Were you satisfied with the therapies?

d. Why were you satisfied, or why not?

5. What changes in your symptoms or overall well-being have you noticed while undergoing the therapies?

a. What do you attribute these changes to?

b. What have you benefited from?

c. What was helpful?

d. What impacts of the therapies have you noticed on your overall well-being?

e. Do you feel like anything else in your life has changed under the therapy?

f. **If not**, how do you feel about that? What do you think: why were there no changes?

Assessment of the Effectiveness and Risks of Complementary Therapies

6. How would you assess the risks of the therapies you used?

a. What dangers do you see in the application of these therapies?

b. How would you assess the effectiveness of these therapies?

Re-application of Complementary Therapies

7. Would you use these therapies or something else again?

a. How might your attitude towards the therapies have changed during their use?

Would you like to add anything else?

I sincerely thank you for the conversation!
